# Supplementary material for: Epidemiology of canine ehrlichiosis and molecular characterization of Erhlichia canis in Bangladeshi pet dogs
Source: PLoS One. 2024 Dec 5;19(12):e0314729. doi: 10.1371/journal.pone.0314729 (PMC11620671; doi:10.1371/journal.pone.0314729)
Supplement: S1 File — (DOCX) [file pone.0314729.s001.docx]

| Epidemiology of Canine Ehrlichiosis and Molecular Characterization of *Erhlichia Canis* in Bangladeshi Pet Dogs |
| --- |
| 1. **Owner's Name:** Date - 2. District- Division- 3. Mobile no: 4. Sex- Male / Female 5. Age - ≤ 12 month /13 - ≤ 24 month/≥ 25 month 6. Breed- Local / Exotic 7. Month- 8. Presence of tick (on the dog's body) - Yes / No 9. Outdoor access of dogs - Yes / No 10. Regular ectoparasitic treatment of dogs - Yes / No 11. Living area of dogs- Rural/ Urban |
