# Supplementary material for: Epidemiology of canine ehrlichiosis and molecular characterization of Erhlichia canis in Bangladeshi pet dogs
Source: PLoS One. 2024 Dec 5;19(12):e0314729. doi: 10.1371/journal.pone.0314729 (PMC11620671; doi:10.1371/journal.pone.0314729)
Supplement: Attachment — Submitted filename: Response to Reviewers.docx [file pone.0314729.s004.docx]

The authors sincerely thank the reviewers for their constructive comments, which greatly improved the scientific content and presentation of the paper. All changes made in response to their suggestions are highlighted as track changes in the revised manuscript.

**Reviewer #1:** I appreciate your response and the thoughtful way the comments were addressed. However, the supplementary materials were not cited in the main text. I recommend that the authors cite the supplementary files "Supplementary 1.docx" and "Erlichia Supplementary.xlsx" in the appropriate sections of the main text. Additionally, please remove "Supporting Information Fig. 2 raw.tif.

**Response:** Thank you for your valuable feedback. We have cited the supplementary files ("Supplementary 1.docx" and "Erlichia Supplementary.xlsx") in the relevant sections of the main text as suggested, and we have also removed "Supporting Information Fig. 2 raw.tif" as recommended.names throughout the manuscript, adhering strictly to the journal’s guidelines for formatting.

**Reviewer #3**: Line 54 - "E. canis", scientific genus name at the beginning of a sentence should be written in full.

**Response:** Thank you for bringing this to our attention. We've gone through the manuscript and made sure that "E. canis" is written in full as Ehrlichia canis at the beginning of sentences.

Line 93 – correct “… in south a sian countries…”

**Response:** Corrected

Item 2.8 Statistical analysis also contains mortality
“…The mortality and case fatality was calculated based on the follow-up communication with the dog owners. Z-tests for proportions were used to compare prevalence, mortality, and case fatality among districts [24, 25].

**Response:** Thank you for your observation. We have made the necessary corrections in the manuscript

Format tables according to the journal's standards. For example, Table 2 has rows without data

**Response:** Thank you for your feedback. We have reformatted the tables to meet the journal's standards and removed any empty rows from Table 2.
