# Supplementary material for: Epidemiology of canine ehrlichiosis and molecular characterization of Erhlichia canis in Bangladeshi pet dogs
Source: PLoS One. 2024 Dec 5;19(12):e0314729. doi: 10.1371/journal.pone.0314729 (PMC11620671; doi:10.1371/journal.pone.0314729)
Supplement: Attachment — Submitted filename: Response to the reviewer.docx [file pone.0314729.s005.docx]

We sincerely thank the reviewers for their valuable feedback and for identifying areas needing further attention. The changes we made in response to their comments are highlighted as tracked changes in the revised manuscript.

Reviewer #3: The authors have made the suggested changes, but should carefully review the entire text, as it still contains information about mortality. For example: line 252 "The present study's overall mortality and case fatality were 3.3 % and 47.1%, respectively"

Response: Thank you for your feedback. We have carefully reviewed the entire text to ensure that all references to mortality statistics have been removed from the manuscript. Please see lines 252–261 for the relevant changes.
